# British Journals

**Published:** 1838

**Authors:** 


### Art. XIV.—British Journals.

We have just received our Edinburgh and London periodicals, down to the beginning of this year; but have neither time nor space for analysis in this number. The Ed. Med. and Surg. Journal, contains several elaborate articles, which, *prima facie*, are of considerable interest; such as a statistical and pathological Report on the Diseases of the Royal Infirmary, Edinburgh, for five years, by Dr. Home; Surgical Cases in the Glasgow Royal Inf., for two years, by Dr. Davidson; Cases of Hysterical Ischuria and Erratic Urination, by Dr. Laycock, Surgeon, of York Hospital; and an Expermental Investigation of the functions of the eighth pair of nerves, by Dr. Reid. These different papers will be analyzed for our July number. The last is an elaborate communication, abounding in new and original experiments.

The Lon. Med. Gaz. and the Lancet present us with the usual variety. Animal Magnetism, we find, continues to excite an interest in London, and these two weeklys are arrayed against each other on that subject, as well as many others. By the bye, Mr.

Hartshorn, of Providence, is out with new documents in support of Clairvoyance. We shall give some account of them in our next number. D.

